# Effects of equal chemical fertilizer substitutions with organic manure on yield, dry matter, and nitrogen uptake of spring maize and soil nitrogen distribution

**DOI:** 10.1371/journal.pone.0219512

**Published:** 2019-07-09

**Authors:** Yuhui Geng, Guojun Cao, Lichun Wang, Shuhua Wang

**Affiliations:** 1 College of Resources and Environment, Jilin Agricultural University, Changchun, China; 2 Institute of Agricultural Environment and Resources Research, Jilin Academy of Agricultural Sciences, Changchun, China; University of Florida, UNITED STATES

## Abstract

In order to maintain high yields and protect the environment, the replacement of chemical fertilizers with organic ones has received increasing attention in recent years. A 2-year field experiment (2015–2016) was carried out to assess the effects of substituting equal amounts of mineral fertilizer with organic manure on the yield, dry matter (DM), and nitrogen (N) uptake of spring maize (*Zea mays* L.) and on the mineral N (N_min_) distribution in the soil profile. The treatments included chemical fertilizer; different amounts of maize straw, cow manure, and chicken manure; and an unfertilized control (CK). Compared with the chemical fertilizer treatments, equal amounts of substitutions with cow manure or chicken manure increased production, and a 25% nutrient substitution resulted in the best yield increase. Straw return had no effect on maize production, and 100% straw return resulted in reduced production. The N accumulation and DM content both exhibited a slow-fast-slow growth trend throughout the various growth stages, and the average N uptake and DM accumulation in response to the treatments followed the order of chicken manure > cow manure > chemical fertilizer > straw return > CK. The N_min_ content in the profile not only increased as the N_min_ application rate increased but also showed greater increases at certain depths than at the surface, indicating that excessive N led to leaching. These results suggest that an appropriate proportion of organic substitution not only provides enough nutrients but also improves the soil environment and leads to increased yields. This technique represents a practical method of continuously increasing production and reducing the risk of N leaching.

## Introduction

Increases in grain yield over recent decades in China were largely dependent on heavy investments in fertilizer. China has been the world’s greatest consumer of N fertilizer since 1985. However, too much chemical fertilizer results in soil degradation and low use efficiency of applied fertilizers (e.g., N) by crops, which leads to considerable losses of N and environmental pollution [1].

Compared to the separate application of chemical fertilizers, the application of manure is beneficial to the soil nutrient balance, soil structure and moisture-holding capacity, and facilitates environmental protection [2, 3]. The application of organic fertilizers represents a good method of maintaining crop yields and soil organic carbon (SOC) reserves [4]. Thus, manure has been applied as a major amendment to maintain soil fertility [5].

Considerable research has focused on the effect of organic fertilizer applications on yield. Duan (2011) showed that the application of manure along with mineral fertilizer over 15 years in China led to high yields [6]. In addition, Diacono and Montemurro [7] performed more than twenty long-term experiments and reported that organic amendments consistently did not reduce crop yields, and Zhang et al (2016) showed that replacing 30% total N fertilizer (250 kg N ha^-1^) with compost (the compost application rate was 3000 kg ha^-1^, which was equal to 60 kg N ha^-1^) is an effective method of increasing the maize yield, N uptake and soil fertility, and reducing N loss [8]. These findings demonstrate that optimum yields can be achieved by management practices that involve alternative sources of N, and N availability can be successfully balanced with crop uptake [9].

Manure-based fertilization could represent an alternative to mineral fertilizer to achieve high maize yields and improve the soil environment and soil quality [10, 11]. Research has shown that using organic-inorganic compound fertilizers can not only decrease the use of chemical fertilizer but also promote the efficiency and sustainability of agricultural ecosystems over long period of time [12]. A five year study showed that the yield and SOC in organic fertilizer treatments increased by 126% and 7%, respectively, compared with those in chemical fertilizer treatments [13]. The application of organic fertilizer can significantly increase the SOC content and nutrients, indicating that the combination of organic fertilizer and inorganic fertilizer is a good fertilization method for improving maize yield and soil quality [14]. Similar studies also identified that the use of organic fertilizer not only increased the content of soil organic carbon (SOC) and nutrients but also enhanced soil physical properties and soil microbial activity [15–17]. Previous researchers [18, 19] have reported that the application of organic fertilizer and chemical fertilizer can improve microbial activity, biomass, and nutrient utilization efficiency compared with the application of chemical fertilizers only. Other studies have shown that the application of manure can increase maize yields and N uptake, decrease NO_3_^-^-N accumulation in the soil, restore crop productivity and sustainability, and reduce the apparent N surplus (apparent N surplus = applied fertilizer N–N uptake in the crop) [17, 20, 21]; thus represents a feasible method of improving the quality of degraded soils.

In most cases, organic manure combined with mineral fertilizer resulted in significant increases in yield primarily because of the increased total nutrient inputs [22, 23]. The nutrient contributions of manure should be estimated and reported because the mismanagement of manure, including incorrect application rates, timing and methods, on agricultural land can result in nutrient losses to the environment (e.g., nitrate leaching, ammonia volatilization, and P loss via runoff), increased soil salinity, and invasion of pathogens and weeds [24, 25]. Additionally, potential benefits of combined applications of manure and mineral N (N_min_) fertilizers should be assessed to recommend complementary fertilizer rates to overcome the initial limited availability of N supplied as cow manure. All of these factors will contribute to optimizing crop productivity while minimizing the environmental impact of fertilizer applications.

Although previous studies have focused mainly on maize yields, limited information is available on the impact of substituting equal amounts of organic fertilizer for chemical N on maize yields and nutrient uptake. Therefore, the main goal of this study was to determine how equal N substitution by manure affects the crop yield, dry matter (DM), N uptake of spring maize, and the accumulation of N_min_ in the soil through field experiments.

## Materials and methods

### Field experiment site description

The field experiment was performed in 2015 and 2016 at the Experimental Station of Jilin Agricultural University (43°49′6.6″N, 125°23′56.4″E) in Changchun City, which is located in the middle of Jilin Province. This province lies in a humid region of northeastern China, and the mean temperature and annual precipitation of the experimental site is 6.7 °C and 600–700 mm, respectively; the maximum temperature is 35 °C; minimum temperature is -28 °C; and sunshine time is 2,688 hours annually. The annual average effective accumulated temperature is 2900 °C, and the frost-free period is 130–135 days per year. We repeated the same experiment at another location at the Experimental Station of Jilin Agricultural Universityin the second year. The soil type of the experimental field was classified as Luvic Phaeozem, which has a silty loam texture. The initial topsoil properties for organic matter, available N, P and K were 27.4 g kg^-1^, 150.7 mg kg^-1^, 31.2 mg kg^-1^ and 136.1 mg kg^-1^ in 2015, respectively, and 26.1 g kg^-1^, 142.7 mg kg^-1^, 33.5 mg kg^-1^ and 128.6 mg kg^-1^ in 2016, respectively. The soil N and P fertility grade of the experimental land is higher, and the K fertility grade is medium. The SOC content was determined by the K_2_CrO_7_-H_2_SO_4_ oxidation method. The available N was measured by using a micro-diffusion technique after alkaline hydrolysis. The available P was measured by the sodium bicarbonate extraction method. The available K was measured by flame photometry after ammonium acetate(NH_4_Oac) neutral extraction. All methods were described in detail by Lu (2000) [26].

### Experimental design

The experiment involved a randomized block design and was replicated three times. The plots were 6.5 m wide by 9 m long and contained 10 rows spaced 65 cm apart, and each plot area measured 58.5 m^2^. There were 14 treatments in the experiment (Table 1).

**Table 1:** Experimental treatments and fertilizer rates.

Treatment	Nutrient content in straw or manure (kg ha^-1^)				Mineral fertilizer (kg ha^-1^)		
	Application amount	N	P_2_O_5_	K_2_O	N	P_2_O_5_	K_2_O
CK	0.0	0.0	0.0	0.0	0.0	0.0	0.0
MF	0.0	0.0	0.0	0.0	240.0	154.0	197.0
S25	3230	16.3	5.6	33.9	223.7	148.4	163.1
S50	6450	32.5	11.3	67.7	207.5	142.7	129.3
S75	9680	48.8	16.9	101.6	191.2	137.1	95.4
S100	12900	65.0	22.6	135.5	175.0	131.4	61.6
CM25	6560	60.0	38.4	49.2	180.0	115.6	147.8
CM50	13110	120.0	76.8	98.4	120.0	77.2	98.6
CM75	19670	180.0	115.2	147.6	60.0	38.8	49.4
CM100	26230	240.0	153.6	196.8	0.0	0.0	0.0
PM25	3220	60.0	32.6	25.7	180.0	121.4	171.3
PM50	6430	120.0	65.2	51.4	120.0	88.8	145.6
PM75	9650	180.0	97.9	77.2	60.0	56.1	119.8
PM100	12870	240.0	130.5	102.9	0.0	23.5	94.1

Each treatment was designed in accordance with the principle of equalizing the total N content in which different amounts of straw and animal manure returned to the field were calculated on the basis of equal N amounts (240 kg ha^-1^ N, the amount of N applied was determined according to the traditional N applied by local farmers). The amount of P and K applied to each treatment was equal to the amount of P and K (154 kg ha^-1^ P_2_O_5_, 197 kg ha^-1^ K_2_O) contained in the straw or animal manure, The P rate is higher than normal because the N, P, K content in the 100% cow manure treatment was used as the fertilization standard. Moreover, the proportion of P in cow manure was high; therefore, the P application rate of all treatments was higher. Deficient nutrients in organic manure were compensated for by the application of N, P, K fertilizer, and the total nutrient content was as follows: 240 kg ha^-1^ N, 154 kg ha^-1^ P_2_O_5_, and 197 kg ha^-1^ K_2_O.

The maize straw return treatments involved 4 application rates (25%, 50%, 75% and 100% of the total straw), and the straw was chopped into pieces that were approximately 2–4 cm in length before application. The cow manure and chicken manure treatments involved 4 application rates (25%, 50%, 75% and 100% of the total amount of N (240 kg ha^-1^)), and deficiencies in the nutrient contents within the manures and straw were compensated by chemical fertilizer. The cow manure, chicken manure or CK treatments did not receive any straw. The CK treatments received no N, P or K and no straw or manure. We did not add P and K to the control because the control was also used for studying P and K, which was not shown in this paper. The experimental treatments were as follows:

Control (CK): no fertilization and no straw or manure;
MF: mineral NPK fertilizer (240 kg ha^-1^ N, 154 kg ha^-1^ P_2_O_5_, 197 kg ha^-1^ K_2_O);
S25: 25% straw return (containing 16.3 kg ha^-1^ N) + (N_min_ at 223.7 kg ha^-1^);
S50: 50% straw return (containing 32.5 kg ha^-1^ N) + (N_min_ at 207.5 kg ha^-1^);
S75: 75% straw return (containing 48.8 kg ha^-1^ N) + (N_min_ at 191.2 kg ha^-1^);
S100: 100% straw return (containing 65.0 kg ha^-1^ N) + (N_min_ at 175.0 kg ha^-1^);
CM25: 25% cow manure (containing 60 kg ha^-1^ N) + 75% N_min_ (180 kg ha^-1^);
CM50: 50% cow manure (containing 120 kg ha^-1^ N) + 50% N_min_ (120 kg ha^-1^);
CM75: 75% cow manure (containing 180 kg ha^-1^ N) + 25% N_min_ (60 kg ha^-1^);
CM100: 100% cow manure (manure N 240 kg ha^-1^ N);
PM25: 25% chicken manure (containing 60 kg ha^-1^ N) + 75% N_min_ (180 kg ha^-1^);
PM50: 50% chicken manure (containing 120 kg ha^-1^ N) + 50% N_min_ (120 kg ha^-1^);
PM75: 75% chicken manure (containing 180 kg ha^-1^ N) + 25% N_min_ (60 kg ha^-1^); and PM100: 100% chicken manure (containing 240 kg ha^-1^ N).

Forty percent N was applied before sowing as a basal fertilizer, 30% N was applied at the V6 stage (six expanded leaves), and 30% N was applied at the pretasseling phase as topdressing. All the K, P, straw and animal manure were applied as basal fertilizers. The N, P, K, maize straw and animal manure application rates of different treatments are described in Table 1.

The mineral N, P, and K fertilizers were applied before sowing, and urea, superphosphate and potassium sulfate (considering that the sulfur content was less than 20% in the fertilizer, the total amount of sulfur was less, and the difference was smaller when all treatments were applied; hence, the effect of sulfur was ignored here) served as the N, P and K, respectively. The maize straw contained 0.72% N, 0.25% P and 1.50% K (30% moisture content at time of application), and the cow manure contained 1.50% N, 0.96% P and 1.23% K (39% moisture content at time of application). The chicken manure contained 2.87% N, 1.56% P and 1.68% K (35% moisture content at time of application). The application rate was determined based on the total N divided by the N content in manure on a dry weight basis.

The maize hybrid variety XY335 was planted. This variety is bred by the American Pioneer Co., and it has a compact plant type, exhibits mid-late maturation, and requires 127 days from emergence to reach maturity. The plots were overseeded and thinned during the seedling stage, and the final plant density was approximately 65000 plants ha^-1^. The seeds were sown on 3 May 2015 and 3 May 2016 and harvested on 29 September 2015 and 2 October 2016, respectively. Weeds were controlled by preemergence herbicides, and no pesticides were applied to the maize plants. Weed control is mainly achieved by applying pesticides and herbicides and performing artificial weeding.

### Crop harvest, plant and soil sampling, and analyses

Fresh material from three representative plants from each plot was randomly sampled at the ground level at each of the following growth stages: V3, V6, V12, VT, R2, R3, R5, and R6 (18, 47, 64, 76, 96, 111, 121 and 131 days after germination in 2015, respectively, and 17, 44, 62, 77, 98, 114, 124 and 135 days after germination in 2016, respectively). The plant samples were separated into four components: 1) stalks (including stems, leaf sheaths and tassels), 2) leaves, 3) husks + cobs, and 4) grain. All the samples were heated at 105 °C for 30 minutes (to stop the action of enzymes and prevent the decomposition of active substances or other components) and then dried to a constant weight at 70 °C [27]. Each plant fraction was weighed to obtain its DW and then ground into a fine powder by a hammer mill which was then passed through a 2-mm mesh screen [28]. Appropriate amounts of ground plant DM were used to determine the N concentration. The N content was analyzed by the micro-Kjeldahl procedure after the samples were digested with H_2_SO_4_-H_2_O_2_ [26]. The N accumulation (kg ha^-1^) in the plant fractions was calculated by multiplying the N concentration fraction (%) by the DM fraction (kg ha^-1^). Soil samples were collected from the top 100 cm of the soil profile (at 20-cm depth intervals). The soil N_min_ (NH_4_^+^-N + NO_3_-N) concentration was determined by the continuous flow analysis technique (TRAACS 2000, Bran and Luebbe, Norderstedt, Germany).

At maturity, 10 m^2^ of maize was manually harvested from the middle of each plot to determine the grain yield, which was adjusted to a 14% moisture content. In addition, the 1000-grain weight and grain number per ear were determined.

### Statistical analysis

Homogeneity of variance and normality tests were performed for each variable analyzed. An analysis of variance was used to compare the grain yield, DM, N uptake, and soil NO3-N content among treatments using the general linear model (GLM) in SPSS 17.0 (SPSS Inc., Chicago, USA). Differences were compared using Duncan’s multiple comparison at the 0.05 level of probability.

## Results

### Grain yield and DM accumulation

As shown in Table 2, the yield of all fertilization treatments was higher than that of CK, and the yield was influenced by the different fertilization treatments under the same total NPK inputs. The yield in response to straw return increased with increasing straw return but decreased when 100% straw was returned. Significant differences in the yield were not observed in S75, S50, S25 and MF treatments, however the yield of MF was significantly higher than that of the S100 treatment. The yield in response to the substitution of chemical fertilizer with CM decreased with increasing amounts of CM. The average yield of CM25 combined with 75% chemical N in two years was 11.9% higher than that with CM100. In terms of yield, the partial substitution of N fertilizer with CM was better than the 100% substitution with CM. The yield of PM substitution also decreased with increasing amounts of PM. The yield in response to PM25 combined with 75% chemical N was the highest, and the grain yield was approximately 9.8% higher in the PM25 treatment than the MF treatment and 14.0% higher in the PM25 treatment than the PM100 treatment. These findings mean that in terms of maize yield, the partial substitution for N fertilizer with PM was better than MF only or 100% substitution with PM. The mean grain yield during the 2 years of field experiments significantly differed among the treatments and years; the treatment-by-year interaction was also significant for yield (Table 2).

**Table 2:** Grain yields in 2015 and 2016 (kg ha^-1^).

Treatment	2015	2016
CK	5654±255 g	5737±658 f
MF	8357±508 cde	8875±562 bcd
S25	8175±526 de	8614±632 cde
S50	8235±671 de	8747±607 bcde
S75	8402±582 bcde	8733±480 bcde
S100	7432±492 f	8081±546 e
CM25	8743±614 abcd	9080±475 abc
CM50	8515±609 bcd	8941±672 abcd
CM75	8111±600 def	8617±454 cde
CM100	7671±642 ef	8255±672 de
PM25	9331±708 a	9598±454 a
PM50	9055±694 abc	9408±652 ab
PM75	9121±232 ab	9077±734 abc
PM100	8053±704 def	8556±422 cde
Source of variation		
Treatment (T)	***	
Year (Y)	**	
T × Y	***	

Different letters indicate significant differences at the P < 0.05 level. NS, not significant (P > 0.05); *, **, and *** indicate significance at P < 0.05, 0.01, and 0.001, respectively.

**Fig 1:**
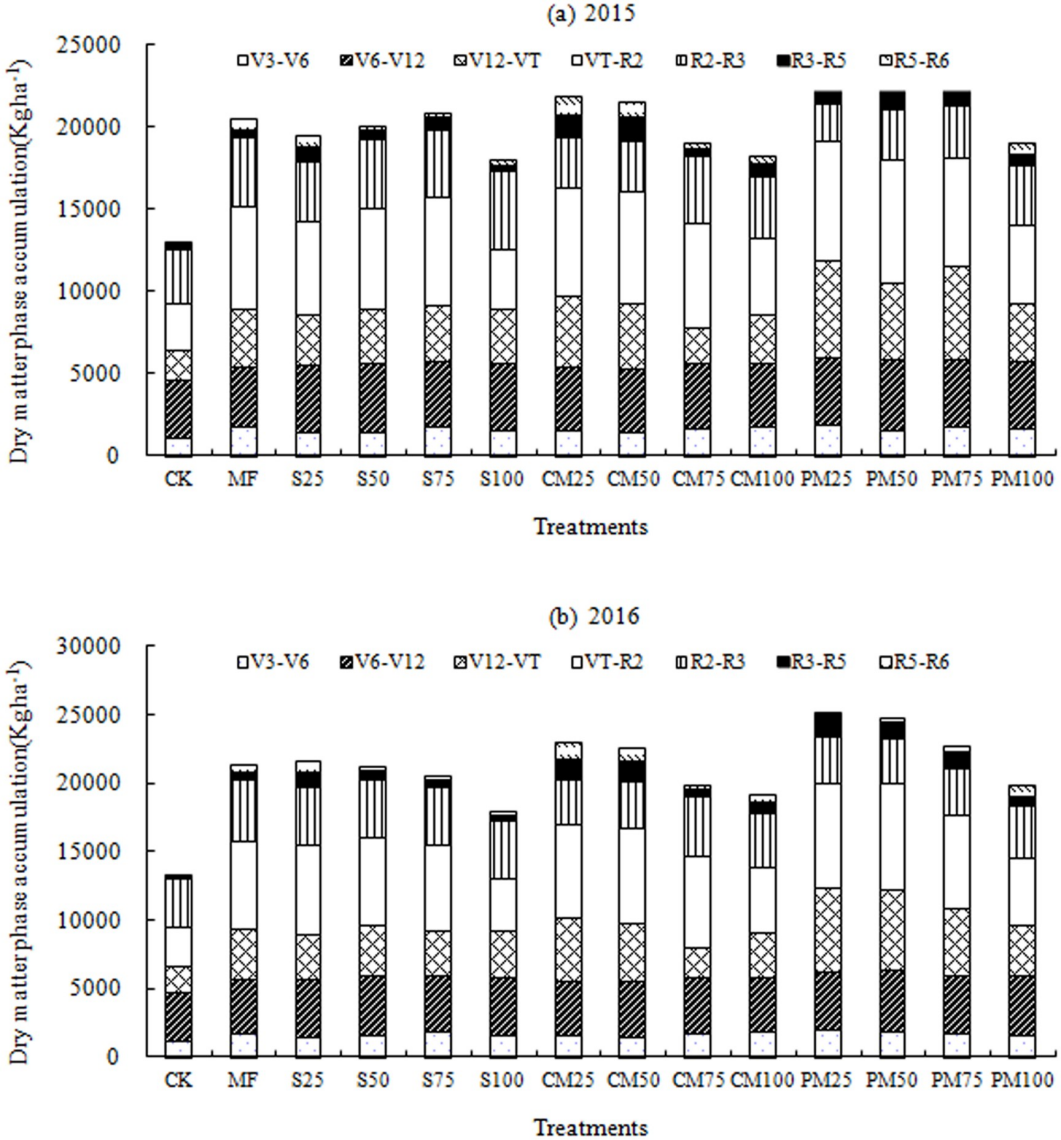
Dry matter phase accumulation of spring maize in response to different amounts of manure application.

The DM accumulation of spring maize varied significantly (p<0.05) between treatments (Table 3). The DM accumulation was slow at first but rapidly increased after the V6 stage along with the rapid growth of the maize plants, although it slowed again after the R2 stage until maturity. Thus, the accumulation exhibited a slow-fast-slow growth trend during the course of the various growth stages (Fig 1). At maturity, the average DM accumulation followed the order of PM > CM > MF > straw return > CK. The highest amount of DM was recorded in PM25, which was 9.0% higher than that in the MF treatment and 67.9% higher than that in CK. The lowest amount of DM in the fertilization treatment was observed in S100, which was 3.1% lower than that in MF but still 49.3% higher than that in CK. The maize DM decreased as the amount of substituted CM or PM increased, although the DM in response to straw return did not differ except for the S100, in which it had the lowest DM among the fertilization treatments.

**Table 3:** Dynamic changes in N accumulation of the maize and N accumulation variance analysis results during different growth stages.

Growth stages		VE-V3		V3-V6		V6-V12		V12-VT		VT-R2		R2-R3		R3-R5		R5-R6		Vegetative stages		Reproductive stages	
Year		2015	2016	2015	2016	2015	2016	2015	2016	2015	2016	2015	2016	2015	2016	2015	2016	2015	2016	2015	2016
Stage accumulation (kg ha^-1^)	CK	0.9	1.0	14.5	14.7	17.1	17.4	23.4	24.5	16.9	17.3	10.0	10.3	0.8	0.8	2.8	2.9	55.9	57.7	30.4	31.3
	MF	1.0	1.1	30	30.4	58.2	61.1	49.9	52.4	25.7	26.8	9.9	10.4	11.6	12.2	1.8	1.9	139.2	144.9	49.1	51.3
	S25	1.0	1.0	24.6	25.4	60.8	62.6	23.8	25.9	43.7	49.3	11.9	14.2	6.4	7.1	3.4	3.9	110.1	114.8	65.4	74.4
	S50	1.0	1.0	28.2	29.6	62.7	65.8	30.1	33.6	38.5	40.0	10.9	11.4	8.8	9.2	4.4	4.6	122	130.1	62.5	65.2
	S75	1.1	1.1	33.0	33.4	64.5	67.4	32.7	32.0	37.8	35.3	11.1	11.5	6.8	4.9	1.6	1.7	131.2	134	57.4	53.4
	S100	0.5	0.6	31.7	32.0	57.5	59.5	29.8	31.3	35.2	37.2	5.4	4.8	7.1	7.4	5.0	5.3	119.6	123.3	52.7	54.7
	CM25	0.7	0.8	31.0	31.2	74.9	78.7	42.9	45.4	26.1	27.1	7.3	7.8	7.7	8.2	13.5	14.3	149.6	156.1	54.5	57.5
	CM50	1.0	1.1	29.8	31.3	70.0	73.5	45.4	47.0	24.6	25.6	6.0	6.4	5.3	5.7	13.0	13.9	146.3	152.9	48.9	51.6
	CM75	1.2	1.3	28.1	28.4	67.6	70.1	45.9	48.2	19.1	19.8	12.3	12.9	3.2	3.6	13.2	13.9	142.8	148	47.8	50.2
	CM100	0.8	0.9	25.8	26.8	67.3	71	42.4	45.1	17.7	18.4	9.0	9.4	10.4	11.1	4.9	5.2	136.2	143.7	42.1	44.1
	PM25	0.9	0.9	33.6	34.1	78.2	82.7	43.6	45.3	28.6	29.9	11.0	16.4	9.2	9.7	12.7	13.7	156.2	163	61.5	69.7
	PM50	0.9	0.9	32.9	37.8	75.5	78.9	39.4	50.1	27.3	28.4	9.2	9.7	14.1	14.9	4.6	4.8	148.7	167.8	55.2	57.8
	PM75	1.2	1.2	30.3	28.4	75.7	79.4	35.9	31.2	25.0	26.1	10.6	11.1	18.4	19.1	3.0	3.1	143	140.2	57.0	59.4
	PM100	1.0	1.1	29.2	29.0	71.7	74.6	31.5	32.8	28.4	29.6	4.9	5.2	18.5	19.8	1.7	1.9	133.4	137.5	53.6	56.5
Source of variation	Treatment (T)	***		***		***		***		***		***		***		***		***		**	
	Year (Y)	***		***		***		***		***		***		***		***		***		***	
	T × Y	NS		***		**		***		***		***		***		**		***		***	

"Vegetative stages" refers to the stages from VE to VT, and "Reproductive stages" refers to the stages from VT to R6. NS, not significant (P > 0.05); *, **, and *** indicate significance at P < 0.05, 0.01, and 0.001, respectively.

### Changes in N accumulation during different growth stages

The N accumulation also exhibited a slow-fast-slow growth trend over the course of the various growth stages. The accumulation was initially slow, increased rapidly with the growth of maize, and then slowed again after the R2 stage. At the end of the growing season, the N accumulation during the vegetative stage was higher than that during the reproductive stage in all treatments, indicating that the spring maize accumulated more N during the vegetative stage than during the reproductive stage (Table 3). The maximum accumulation in all treatments occurred between stages V6 and V12. The average N accumulation in response to the treatments followed the order of PM > CM > MF ≈ straw return > CK. The highest N accumulation occurred in treatment PM25 and reached 78.2 and 82.7 kg ha^-1^ between the VT and R2 stages in 2015 and 2016, respectively (Table 3); these values were 34.4% and 35.3% higher than those in treatment MF, respectively. The treatment and year effects at both the vegetative stage accumulation and reproductive stage accumulation significantly differed among the treatment; the treatment-by-year interaction was also significant except in the VE-V3 stages (Table 3).

### Mineral N (N_min_) concentration and distribution in the soil profile

**Fig 2:**
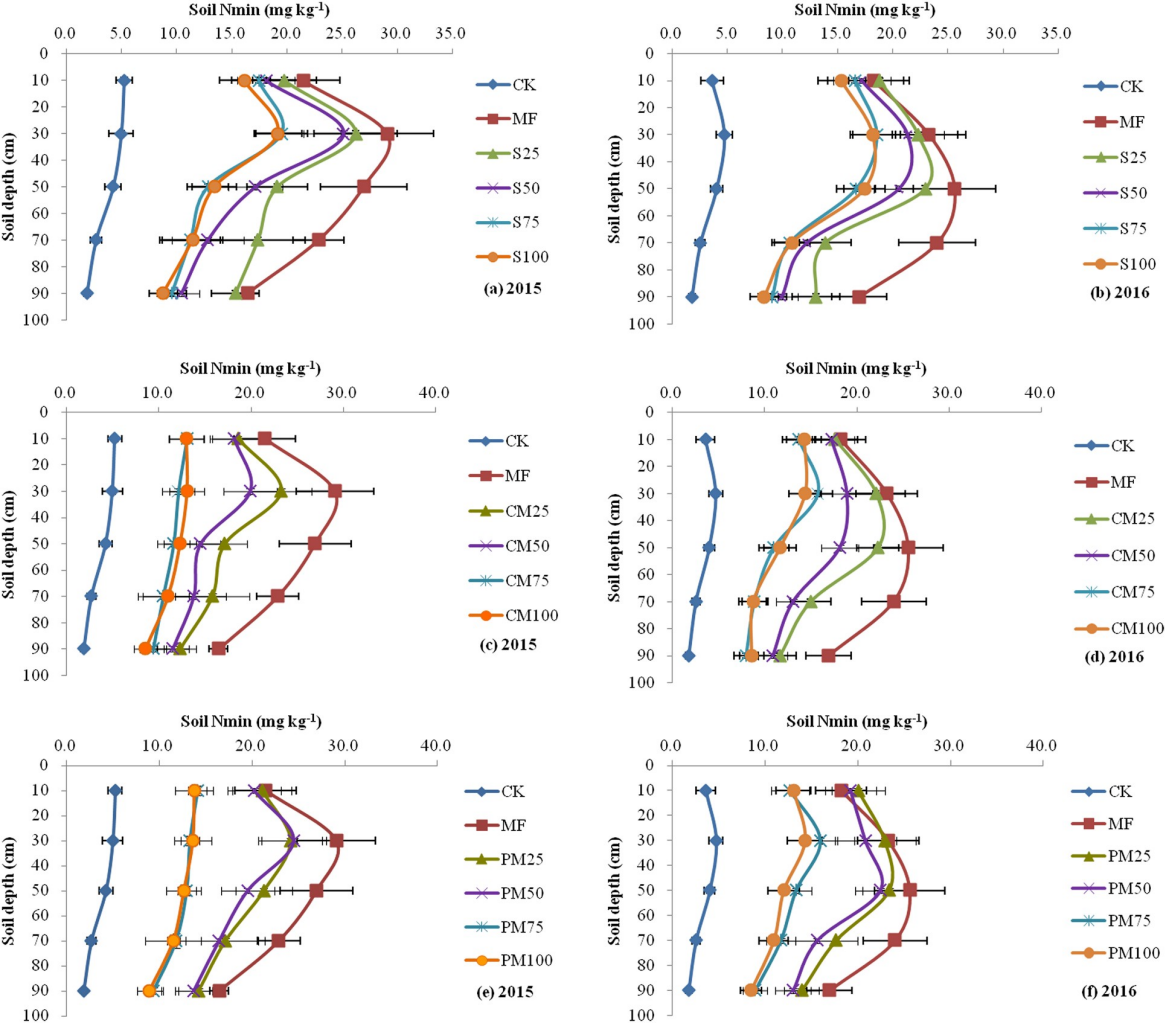
Mineral N concentration and distribution in the soil profile. The vertical bars represent the standard errors of the data.

At harvest, the N_min_ content in the profile generally increased with the increase in MF application, and the soil N_min_ content in the fertilized treatments varied significantly across the two years (Table 4). Overall, The MF treatment resulted in the highest N_min_ content, and the CK resulted in the lowest N_min_ content within the soil profile (Fig 2). The measurement of the N_min_ content along the soil profile down to 100 cm showed that the N_min_ contents in the CK, S100, CM100, PM100 and S75 CM75, PM75 decreased as the profile depth increased; meanwhile, there was an additional accumulation in the soil (20–40 cm in 2015 and 40–60 cm in 2016) compared with that at the surface or other depths of N_min_ contents in the MF, S50, CM50, PM50, S25, CM25, and PM25; the N_min_ content at 20–40 cm depth was 35.5% higher than that at the surface in 2015; and the N_min_ content at the 20–40 cm depth was 40.4% higher than that at the surface in 2016. The additional accumulation in the soil indicated that excessive N_min_ fertilizer leads to leaching.

**Table 4:** Statistically significant differences in mineral N concentration and distribution in the soil profile.

Soil depth (cm)		0–20		20–40		40–60		60–80		80–100	
Year		2015	2016	2015	2016	2015	2016	2015	2016	2015	2016
Soil N_min_ (mg kg^-1^)	Straw return										
	CK	e	d	d	d	e	e	e	e	e	e
	MF	a	a	a	a	a	a	a	a	a	a
	S25	b	a	b	ab	b	b	b	b	b	b
	S50	c	b	b	b	c	c	c	c	c	c
	S75	c	b	c	c	d	d	d	d	cd	cd
	S100	d	c	c	c	d	d	d	d	d	d
	CM										
	CK	d	c	e	e	e	e	e	e	d	d
	MF	a	a	a	a	a	a	a	a	a	a
	CM25	b	a	b	a	b	b	b	b	b	b
	CM50	b	a	c	b	c	c	c	c	b	b
	CM75	c	b	d	c	d	d	d	d	c	c
	CM100	c	b	d	d	d	d	d	d	c	c
	PM										
	CK	d	d	d	e	e	d	d	e	d	d
	MF	a	a	a	a	a	a	a	a	a	a
	PM25	ab	ab	b	a	b	b	b	b	b	b
	PM50	b	b	b	b	c	b	b	c	b	b
	PM75	c	c	c	c	d	c	c	d	c	c
	PM100	c	c	c	d	d	c	c	d	c	c

Different letters indicate significant differences at the P < 0.05 level.

## Discussion

### Grain yield and DM accumulation

In recent years, an increasing number of studies have focused on manure nutrients, and increased grain yields of 27% in response to NPK + Azolla compost applications compared with the control [29] have been reported. Manure application alone or combined with NPK resulted in significant increases in maize yields and sustained growth of maize yields over the long term [14]. However, Xin et al. [30] reported that MF is very important for raising crop yields, and the complete substitution of MF with organic fertilizer (where N fertilizer was 100% replaced by compost, and to match the same rate of NPK, chemical P and K fertilizers were supplied to the compost) resulted in decreased production. Edmeades [31] reported no significant difference in the long-term effects on crop production between MF and manure, and crop yields obtained from farmyard manure were 9.5% lower than that obtained from mineral fertilizers applied at similar N rates [32].

In the present research, the yield was significantly influenced by the different fertilization treatments under similar nutrient inputs. First, the yield in all straw return treatments was higher than that in the CK treatment, and the effects of the different organic manure returns on yield were different. The effects of straw return on maize yield followed the order of S75 > MF > S50 > S25 > S100 > CK (Table 2). The yield in response to straw return increased with increasing straw return but decreased at S100. The yield in most of the treatments involving straw return was lower than that in response to the MF treatment, and only the yield in S75 was slightly higher than that in the mineral fertilizer treatment. Except for yields of all treatments being significantly higher than those of the S100 treatments, there was no significant difference between the yields of the fertilizer and straw return treatments.

One possible reason may be that too much straw return not only reduced the amount of available nutrients but also consumed more N for microbial decomposition; moreover, too much straw return may have generated too many macropores and affected the physical character of the soil. In northern China, soil moisture is very important in spring, and too much straw tends to make the soil too loose and facilitates water loss, which is also an important reason for why farmers are unwilling to return straw to the field.

This trend was different for CM and PM, and the yield in response to CM and PM substitution decreased with increasing amounts of applied manure. One reason for this result may be due to the reduced amount of chemical N applied, which resulted in a lack of available nutrients. MF nutrients are known to be taken up by crops immediately after application [33, 34], while nutrients derived from manure must first be mineralized and then transformed into forms that can be taken up by crops. The nutrients released from organic fertilizers slowly cannot be detected in the short run [35]. Furthermore, due to the high content of P and K in the manure, the amount of P and K applied was 154 kg ha^-1^ P_2_O_5_ and 197 kg ha^-1^ K_2_O, respectively; when MF was 100% replaced by CM, excessive organic substitution of nitrogen fertilizer will lead to accumulation of P and K in the soil, increasing the risk of environmental pollution. Research [29] also confirmed that the effects of different organic amendments on soil microbial biomass C, SOC fractions and degree of humification varied and the application of Azolla compost in combination with inorganic fertilizers resulted in more passive fractions of soil C than cow dung, rice husk dust and green manure. The N content of cow dung, green manure, Azolla compost and rice husk dust was 1.59, 2.67, 1.52 and 1.84%, respectively. The N_min_ content in a compost+NPK+straw treatment was more variable and higher on average than in the inorganic treatment in three years [8, 29]. Li’s research [36] found that organic and inorganic fertilizers have different effects on maize yields at different times, with inorganic fertilizer capable of rapidly increasing maize yields in the same year and organic fertilizers capable of increasing maize yields in subsequent years. Our study was consistent with that of former studies, suggesting that MF could be partially substituted by organic fertilizer to increase yields. The average yield in response to the different treatments followed the order of PM > CM > MF > straw return. These results could be due to the positive effects of manure rather than the N supply. Organic fertilizers typically release nutrients (macro- and micronutrients) gradually and supply crops throughout their growth period [37]. Manure also increased the soil organic matter (SOM) content, improved the soil physical and chemical properties, and stimulated soil microorganism activities [38, 39]. Therefore, appropriate organic substitution under equal N conditions is a good method of maintaining the soil nutrient balance; when nutrient supply is adequate, it is important to create a good soil environment by applying organic fertilizers for higher yields. However, excessive organic substitution of N fertilizer will lead to not only yield reduction but also P and K build-up in soil and endangering the environment.

The DM accumulation exhibited a slow-fast-slow growth trend during the course of the various growth stages. Because of the relatively slow growth of Maize at seedling stage, the accumulation of dry matter was less. There was a fast grow period at jointing stage, the accumulation of dry matter began to increase rapidly, and then began to increase slowly at heading stage. The DM accumulation in response to the treatments followed the order of PM > CM > MF > straw return > CK. Similar to yield, the straw return treatment exhibited slightly different results, with the DM decreasing with increasing straw return and manure. An increase in straw return corresponded to a decrease in MF application, and the slow decomposition of straw affected the DM accumulation because of the reduced supply of nutrients, especially N. Because straw decomposes slowly and the nutrient supply was mainly affected by the application of MF, the DM accumulation was determined mainly by the amount of MF applied.

### Changes in N accumulation during different growth stages

Nitrogen uptake is influenced by different fertilization methods and plays an important role in maize growth and grain yield. Some investigations have found that when organic and MF are applied separately, the crop takes up a greater proportion of N from inorganic fertilizer than from legume residue (40% versus 17% of input respectively)[40–42]. Inorganic fertilizers contribute a large amount of available N upon application, while legume residues show a delayed and sustained release of N [43, 44]. However, other studies have demonstrated that manure applications significantly increased maize grain yields, the N uptake of maize without manure was reported to be lower than that with manure and the mean N uptake by maize was 1.3 g plant^−1^ for the treatments without manure and 2.4 g plant^−1^ for the treatments with manure [20]. In the present study, the average N accumulation in response to the treatments followed the order of PM > CM > MF ≈ straw return > CK. The aboveground biomass accumulation and N uptake rate exhibited similar increasing trends during the growth period. Previous studies have shown that the N uptake rates in straw- and compost-amended treatments were higher than in the MF treatments in the second year [8], demonstrating that N uptake can be increased by manure applications. Similar to this study, MF was partially replaced by organic fertilizer in these studies; unlike this study, the study conducted by Zhang et al. (2016) [8] did not apply equaly N substitution. The highest N accumulation occurred in PM25, which presented values that were 34.4% and 35.3% higher than that in the MF treatment between the VT and R2 stages in 2015 and 2016, respectively (Table 3). Zhang’s research also found that the partial organic substitution of N fertilizer is a good method for increasing N uptake, maize yields, soil fertility and reducing N losses, probably because the organic fertilizers release nutrients (macro- and micronutrients) gradually and supply the crop throughout the growing period [8]. Zhang et al. (2016) showed that 30% N fertilizer was replaced by cow waste, but the total N was reduced compared with MF; compare to this study where 25% replacement and equal N substitution was used. These results demonstrated that partial organic substitution of N is conducive to N uptake.

### Mineral N (N_min_) concentration and distribution in the soil profile

Excessive N_min_ residue in the soil after harvest corresponds to an increase in potential leaching, which can cause N losses and groundwater pollution. Research has shown that more than half of the applied N fertilizer is lost to the air as gas emissions or into the groundwater as leachate, with less than half of the N fertilizer absorbed by crops [45]. Previous research has illustrated that manure helps maintain stable N, P and SOM contents in the topsoil up to four years. The results of a long-term organic manure project provided evidence that the soil quality and fertility simultaneously improved as the SOM contents increased, which provided a solid foundation for sustainable soil productivity [46, 47] by preventing soil nutrients from leaching and maintaining residual compounds in the topsoil after rapid mineralization [48, 49].

In this study, although all treatments had equal N doses, the N_min_ content in the profile increased when the MF application rates increased. In all treatments, the N_min_ content of MF was the highest. The content of N_min_ decreased with increasing organic manure substitution. The content of N_min_ in the CM100, PM100, S100, CM75, PM75, and S75 showed no difference in the soil profile but was still higher than that in the no fertilizer (CK) treatment. Previous research has confirmed that N applied with P and/or manure could reduce soil NO_3_^-^-N accumulation [50, 51]. Moreover, the concentration of soil N_min_ in the 0 to 100 cm soil layer increased significantly under increases in N levels when only N fertilizer was applied but increased slightly when N fertilizer was applied with P fertilizer and/or manure [20]. The main reason may be due to the higher N uptake than the treatments without manure, indicating that manure application reduced the potential risk of NO_3_^-^-N leaching. This finding suggested that the application of organic chemical is beneficial for maintaining the soil nutrient balance and improving the soil physical properties and contributes to environmental improvement [2, 3].

Compared to the other treatments, the N_min_ content in the MF, S25, CM25, PM25, S50, CM50, and PM50 was higher at depths of 20–40 cm (in 2015) or 40–60 cm (in 2016) than at the surface or other depths. One possible reason maybe there is more rainfall in 2016 than in 2015, so nitrogen leaching is deeper in 2016 than that in 2015. The high accumulation of N at harvest was mainly because of the high N_min_ fertilizer input rate and not the total N input. The total N application rate was the same, indicating that excessive N_min_ fertilizer leads to leaching. Previous research [11] has shown that a mixed application of 30 Mg ha^-1^ cattle manure and 100 kg N ha^-1^ MF (the total N applied was an average of 344 kg N ha^−1^ during the 7 years) led to high yields, and the mean N mineralization over 7 years was 98 kg N ha^−1^ when the first growing season was excluded; this is similar to this research, where organic fertilizers were used to replace MF partially to achieve higher yields. However, higher organic fertilizer application did not result in higher residual inorganic N content in the soil, which indicates that the N maintained in the organic form was more difficult to lose than the N_min_ fertilizer [11]. A consequence of increased SOM is that some soil chemical and biological properties are improved [31, 48]. Manure application may increase the proportion of clay particles and aggregates, increase the cation exchange capacity, and increase NO_3_^-^-N immobilization in the soil [52]. An increase in NO_3_^-^-N immobilization upon application of manure in this study may have played an important role in minimizing potentially leachable NO_3_^-^-N accumulation in the soil via soil structure modifications and subsequent root proliferation [20].

## Conclusions

Clear increases in the yield, DM and N uptake were observed under the same N amount with partial manure substitution. However, excessive organic substitution of N fertilizer led to not only yield reduction but also P and K build-up in the soil. These findings indicate that appropriate organic substitution is beneficial to yield, excessive organic fertilizer substitution will lead to insufficient nitrogen and accumulation of phosphorus and potassium, potentially endangering the environment. The effects of different organic substitutions varied depending on the amount of nutrients in the organic fertilizer and the decomposition rate. the N_min_ content in the profile increased with the increase of MF application rates. Excessive N fertilizer did not increase production instead lead to risk of N leaching. Appropriate organic substitution under equal N conditions is a good method of maintaining the soil nutrient balance, improving the soil physical properties and reducing N_min_ fertilizer leaching.

## Supporting information

S1 File Data for the cited Tables 1, 2, 3 and 4, and Figs 1 and 2 in the manuscript. (DOC)
