# Supplementary material for: Effects of equal chemical fertilizer substitutions with organic manure on yield, dry matter, and nitrogen uptake of spring maize and soil nitrogen distribution
Source: PLoS One. 2019 Jul 9;14(7):e0219512. doi: 10.1371/journal.pone.0219512 (PMC6615609; doi:10.1371/journal.pone.0219512)
Supplement: S1 File — (DOC) [file pone.0219512.s001.doc]

**Data for the cited T**able 1. Experimental treatments and fertilizer rates

| Treatment | Nutrient content in straw or manure (kg ha^-1^) | | | | Mineral fertilizer (kg ha^-1^) | | |
| --- | --- | --- | --- | --- | --- | --- | --- |
|  | Application amount | N | P_2_O_5_ | K_2_O | N | P_2_O_5_ | K_2_O |
| CK | 0.0 | 0.0 | 0.0 | 0.0 | 0.0 | 0.0 | 0.0 |
| MF | 0.0 | 0.0 | 0.0 | 0.0 | 240.0 | 154.0 | 197.0 |
| S25  S50  S75  S100 | 3230 | 16.3 | 5.6 | 33.9 | 223.7 | 148.4 | 163.1 |
|  | 6450 | 32.5 | 11.3 | 67.7 | 207.5 | 142.7 | 129.3 |
|  | 9680 | 48.8 | 16.9 | 101.6 | 191.2 | 137.1 | 95.4 |
|  | 12900 | 65.0 | 22.6 | 135.5 | 175.0 | 131.4 | 61.6 |
| CM25  CM50  CM75  CM100 | 6560 | 60.0 | 38.4 | 49.2 | 180.0 | 115.6 | 147.8 |
|  | 13110 | 120.0 | 76.8 | 98.4 | 120.0 | 77.2 | 98.6 |
|  | 19670 | 180.0 | 115.2 | 147.6 | 60.0 | 38.8 | 49.4 |
|  | 26230 | 240.0 | 153.6 | 196.8 | 0.0 | 0.0 | 0.0 |
| PM25  PM50  PM75  PM100 | 3220 | 60.0 | 32.6 | 25.7 | 180.0 | 121.4 | 171.3 |
|  | 6430 | 120.0 | 65.2 | 51.4 | 120.0 | 88.8 | 145.6 |
|  | 9650 | 180.0 | 97.9 | 77.2 | 60.0 | 56.1 | 119.8 |
|  | 12870 | 240.0 | 130.5 | 102.9 | 0.0 | 23.5 | 94.1 |

**Data for the cited Table 2. Grain yields in 2015 and 2016 (kg ha^-1^**)

| Treatment | 2015 | 2016 |
| --- | --- | --- |
| CK | 5654±255 g | 5737±658 f |
| MF | 8357±508 cde | 8875±562 bcd |
| S25 | 8175±526 de | 8614±632 cde |
| S50 | 8235±671 de | 8747±607 bcde |
| S75 | 8402±582 bcde | 8733±480 bcde |
| S100 | 7432±492 f | 8081±546 e |
| CM25 | 8743±614 abcd | 9080±475 abc |
| CM50 | 8515±609 bcd | 8941±672 abcd |
| CM75 | 8111±600 def | 8617±454 cde |
| CM100 | 7671±642 ef | 8255±672 de |
| PM25 | 9331±708 a | 9598±454 a |
| PM50 | 9055±694 abc | 9408±652 ab |
| PM75 | 9121±232 ab | 9077±734 abc |
| PM100 | 8053±704 def | 8556±422 cde |
| Source of variation |  |  |
| Treatment (T) | *** | |
| Year (Y) | ** | |
| T × Y | *** | |

Different letters indicate signiﬁcant differences at the P < 0.05 level. NS, not significant (P > 0.05); *, **, and *** indicate significance at P < 0.05, 0.01, and 0.001, respectively.

**Data for the cited Fig 1. Dry matter phase accumulation of spring maize in response to different amounts of manure application**

| 2015 | V3-V6 | V6-V12 | V12-VT | VT-R2 | R2-R3 | R3-R5 | R5-R6 |
| --- | --- | --- | --- | --- | --- | --- | --- |
| CK | 1127.4 | 3469.6 | 1841.7 | 2816.7 | 3358.3 | 252.8 | 125.0 |
| MF | 1729.9 | 3697.5 | 3538.9 | 6175.0 | 4225.0 | 516.7 | 577.8 |
| S25 | 1441.1 | 4022.1 | 3069.4 | 5741.7 | 3611.1 | 938.9 | 686.1 |
| S50 | 1443.6 | 4234.3 | 3213.9 | 6175.0 | 4152.8 | 563.3 | 289.1 |
| S75 | 1749.0 | 3965.6 | 3394.4 | 6644.4 | 4080.6 | 758.3 | 288.9 |
| S100 | 1528.5 | 4112.2 | 3286.1 | 3611.1 | 4766.7 | 325.0 | 361.1 |
| CM25 | 1498.2 | 3851.9 | 4369.4 | 6536.1 | 3105.6 | 1336.1 | 1191.7 |
| CM50 | 1375.9 | 3942.7 | 3972.2 | 6788.9 | 3120.0 | 1466.1 | 902.8 |
| CM75 | 1634.0 | 4002.3 | 2130.6 | 6427.8 | 4044.4 | 505.6 | 288.9 |
| CM100 | 1727.1 | 3844.5 | 3033.3 | 4582.5 | 3795.3 | 794.4 | 505.6 |
| PM25 | 1887.3 | 4045.7 | 5958.3 | 7222.2 | 2275.0 | 1552.8 | 180.6 |
| PM50 | 1587.8 | 4271.2 | 4694.4 | 7402.8 | 3141.7 | 1083.3 | 252.8 |
| PM75 | 1778.3 | 4041.1 | 5705.6 | 6572.2 | 3213.9 | 1119.4 | 397.2 |
| PM100 | 1606.8 | 4181.1 | 3502.8 | 4694.4 | 3719.4 | 613.9 | 686.1 |

| 2016 | V3-V6 | V6-V12 | V12-VT | VT-R2 | R2-R3 | R3-R5 | R5-R6 |
| --- | --- | --- | --- | --- | --- | --- | --- |
| CK | 1144.0 | 3529.5 | 1933.8 | 2892.5 | 3458.0 | 265.4 | 138.0 |
| MF | 1748.5 | 3882.41 | 3715.8 | 6422.0 | 4446.0 | 527.5 | 617.5 |
| S25 | 1488.5 | 4140.5 | 3337.0 | 6486.5 | 4309.0 | 1040.0 | 773.5 |
| S50 | 1515.8 | 4446 | 3586.5 | 6415.5 | 4348.5 | 591.5 | 303.5 |
| S75 | 1774.5 | 4147 | 3323.0 | 6209.5 | 4218.5 | 538.5 | 303.3 |
| S100 | 1540.5 | 4251 | 3450.4 | 3809.0 | 4205.0 | 341.3 | 390.0 |
| CM25 | 1508.0 | 4044.534 | 4628.0 | 6799.0 | 3289.0 | 1436.5 | 1267.5 |
| CM50 | 1444.7 | 4139.812 | 4108.0 | 7065.5 | 3341.0 | 1566.5 | 968.5 |
| CM75 | 1651.0 | 4153.5 | 2237.1 | 6682.0 | 4246.7 | 564.9 | 303.3 |
| CM100 | 1794.0 | 4056 | 3224.0 | 4745.0 | 3985.0 | 851.5 | 526.5 |
| PM25 | 1917.5 | 4277 | 6194.5 | 7527.0 | 3411.5 | 1638.0 | 195.0 |
| PM50 | 1826.5 | 4465.5 | 5967.0 | 7696.0 | 3328.0 | 1150.5 | 260.0 |
| PM75 | 1670.5 | 4238 | 4959.5 | 6851.0 | 3374.6 | 1163.5 | 403.0 |
| PM100 | 1599.0 | 4348.5 | 3653.0 | 4894.5 | 3905.4 | 656.5 | 754.0 |

**Data for the cited** Table 3. Dynamic changes in N accumulation of the maize and N accumulation variance analysis results during different growth stages

| Growth stages | | VE-V3 | | V3-V6 | | V6-V12 | | V12-VT | | VT-R2 | | R2-R3 | | R3-R5 | | R5-R6 | | Vegetative stages | | Reproductive stages | |
| --- | --- | --- | --- | --- | --- | --- | --- | --- | --- | --- | --- | --- | --- | --- | --- | --- | --- | --- | --- | --- | --- |
| Year | | 2015 | 2016 | 2015 | 2016 | 2015 | 2016 | 2015 | 2016 | 2015 | 2016 | 2015 | 2016 | 2015 | 2016 | 2015 | 2016 | 2015 | 2016 | 2015 | 2016 |
| Stage accumulation  (kg ha^-1^) | CK | 0.9 | 1.0 | 14.5 | 14.7 | 17.1 | 17.4 | 23.4 | 24.5 | 16.9 | 17.3 | 10.0 | 10.3 | 0.8 | 0.8 | 2.8 | 2.9 | 55.9 | 57.7 | 30.4 | 31.3 |
|  | MF | 1.0 | 1.1 | 30 | 30.4 | 58.2 | 61.1 | 49.9 | 52.4 | 25.7 | 26.8 | 9.9 | 10.4 | 11.6 | 12.2 | 1.8 | 1.9 | 139.2 | 144.9 | 49.1 | 51.3 |
|  | S25 | 1.0 | 1.0 | 24.6 | 25.4 | 60.8 | 62.6 | 23.8 | 25.9 | 43.7 | 49.3 | 11.9 | 14.2 | 6.4 | 7.1 | 3.4 | 3.9 | 110.1 | 114.8 | 65.4 | 74.4 |
|  | S50 | 1.0 | 1.0 | 28.2 | 29.6 | 62.7 | 65.8 | 30.1 | 33.6 | 38.5 | 40.0 | 10.9 | 11.4 | 8.8 | 9.2 | 4.4 | 4.6 | 122 | 130.1 | 62.5 | 65.2 |
|  | S75 | 1.1 | 1.1 | 33.0 | 33.4 | 64.5 | 67.4 | 32.7 | 32.0 | 37.8 | 35.3 | 11.1 | 11.5 | 6.8 | 4.9 | 1.6 | 1.7 | 131.2 | 134 | 57.4 | 53.4 |
|  | S100 | 0.5 | 0.6 | 31.7 | 32.0 | 57.5 | 59.5 | 29.8 | 31.3 | 35.2 | 37.2 | 5.4 | 4.8 | 7.1 | 7.4 | 5.0 | 5.3 | 119.6 | 123.3 | 52.7 | 54.7 |
|  | CM25 | 0.7 | 0.8 | 31.0 | 31.2 | 74.9 | 78.7 | 42.9 | 45.4 | 26.1 | 27.1 | 7.3 | 7.8 | 7.7 | 8.2 | 13.5 | 14.3 | 149.6 | 156.1 | 54.5 | 57.5 |
|  | CM50 | 1.0 | 1.1 | 29.8 | 31.3 | 70.0 | 73.5 | 45.4 | 47.0 | 24.6 | 25.6 | 6.0 | 6.4 | 5.3 | 5.7 | 13.0 | 13.9 | 146.3 | 152.9 | 48.9 | 51.6 |
|  | CM75 | 1.2 | 1.3 | 28.1 | 28.4 | 67.6 | 70.1 | 45.9 | 48.2 | 19.1 | 19.8 | 12.3 | 12.9 | 3.2 | 3.6 | 13.2 | 13.9 | 142.8 | 148 | 47.8 | 50.2 |
|  | CM100 | 0.8 | 0.9 | 25.8 | 26.8 | 67.3 | 71 | 42.4 | 45.1 | 17.7 | 18.4 | 9.0 | 9.4 | 10.4 | 11.1 | 4.9 | 5.2 | 136.2 | 143.7 | 42.1 | 44.1 |
|  | PM25 | 0.9 | 0.9 | 33.6 | 34.1 | 78.2 | 82.7 | 43.6 | 45.3 | 28.6 | 29.9 | 11.0 | 16.4 | 9.2 | 9.7 | 12.7 | 13.7 | 156.2 | 163 | 61.5 | 69.7 |
|  | PM50 | 0.9 | 0.9 | 32.9 | 37.8 | 75.5 | 78.9 | 39.4 | 50.1 | 27.3 | 28.4 | 9.2 | 9.7 | 14.1 | 14.9 | 4.6 | 4.8 | 148.7 | 167.8 | 55.2 | 57.8 |
|  | PM75 | 1.2 | 1.2 | 30.3 | 28.4 | 75.7 | 79.4 | 35.9 | 31.2 | 25.0 | 26.1 | 10.6 | 11.1 | 18.4 | 19.1 | 3.0 | 3.1 | 143 | 140.2 | 57.0 | 59.4 |
|  | PM100 | 1.0 | 1.1 | 29.2 | 29.0 | 71.7 | 74.6 | 31.5 | 32.8 | 28.4 | 29.6 | 4.9 | 5.2 | 18.5 | 19.8 | 1.7 | 1.9 | 133.4 | 137.5 | 53.6 | 56.5 |
| Source of variation | Treatment (T) | *** | | *** | | *** | | *** | | *** | | *** | | *** | | *** | | *** | | ** | |
|  | Year (Y) | *** | | *** | | *** | | *** | | *** | | *** | | *** | | *** | | *** | | *** | |
|  | T × Y | NS | | *** | | ** | | *** | | *** | | *** | | *** | | ** | | *** | | *** | |

"Vegetative stages" refers to the stages from VE to VT, and "Reproductive stages" refers to the stages from VT to R6. NS, not significant (P > 0.05); *, **, and *** indicate significance at P < 0.05, 0.01, and 0.001, respectively.

**Data for the cited Fig 2. Mineral N concentration and distribution in the soil profile. The vertical bars represent the standard errors of the data.**

2015

| Nmin | CK | MF | S25 | S50 | S75 | S100 | CM25 | CM50 | CM75 | CM100 | PM25 | PM50 | PM75 | PM100 |
| --- | --- | --- | --- | --- | --- | --- | --- | --- | --- | --- | --- | --- | --- | --- |
| 0-20 | 5.2 | 21.5 | 19.8 | 18.1 | 17.4 | 16.2 | 18.6 | 18.2 | 13.1 | 13.0 | 21.1 | 20.2 | 14.1 | 13.8 |
| 20-40 | 5.0 | 29.1 | 26.2 | 25.1 | 19.5 | 19.2 | 23.2 | 19.9 | 12.1 | 13.1 | 24.1 | 24.5 | 13.3 | 13.6 |
| 40-60 | 4.2 | 27.0 | 19.1 | 17.1 | 12.8 | 13.4 | 17.1 | 14.5 | 11.6 | 12.3 | 21.3 | 19.5 | 12.9 | 12.7 |
| 60-80 | 2.7 | 22.9 | 17.4 | 12.8 | 11.2 | 11.4 | 15.8 | 13.8 | 10.5 | 11.0 | 17.1 | 16.5 | 11.8 | 11.5 |
| 80-100 | 1.9 | 16.5 | 15.3 | 10.4 | 9.5 | 8.8 | 12.3 | 11.5 | 9.4 | 8.6 | 14.3 | 13.7 | 9.4 | 9.0 |

2016

| Nmin | CK | MF | S25 | S50 | S75 | S100 | CM25 | CM50 | CM75 | CM100 | PM25 | PM50 | PM75 | PM100 |
| --- | --- | --- | --- | --- | --- | --- | --- | --- | --- | --- | --- | --- | --- | --- |
| 0-20 | 3.6 | 18.2 | 18.8 | 17.2 | 16.6 | 15.4 | 17.6 | 17.3 | 13.7 | 14.3 | 20.0 | 19.2 | 12.7 | 13.1 |
| 20-40 | 4.7 | 23.3 | 22.3 | 21.3 | 18.5 | 18.2 | 22.1 | 18.9 | 15.8 | 14.4 | 22.9 | 20.8 | 16.0 | 14.3 |
| 40-60 | 4.0 | 25.6 | 22.9 | 20.6 | 16.7 | 17.4 | 22.3 | 18.2 | 11.0 | 11.7 | 23.4 | 22.5 | 13.3 | 12.0 |
| 60-80 | 2.6 | 24.0 | 13.9 | 12.2 | 10.7 | 10.9 | 15.0 | 13.1 | 8.9 | 8.8 | 17.7 | 15.6 | 11.7 | 10.9 |
| 80-100 | 1.8 | 17.0 | 13.0 | 9.9 | 9.1 | 8.3 | 11.7 | 10.9 | 8.0 | 8.6 | 14.0 | 13.0 | 8.9 | 8.5 |

**Data for the cited Table 4. Statistically signiﬁcant differences in mineral N concentration and distribution in the soil profile**

2015

| Nmin | CK | MF | S25 | S50 | S75 | S100 | CM25 | CM50 | CM75 | CM100 | PM25 | PM50 | PM75 | PM100 |
| --- | --- | --- | --- | --- | --- | --- | --- | --- | --- | --- | --- | --- | --- | --- |
| 0-20 | 5.2 | 21.5 | 19.8 | 18.1 | 17.4 | 16.2 | 18.6 | 18.2 | 13.1 | 13.0 | 21.1 | 20.2 | 14.1 | 13.8 |
| 20-40 | 5.0 | 29.1 | 26.2 | 25.1 | 19.5 | 19.2 | 23.2 | 19.9 | 12.1 | 13.1 | 24.1 | 24.5 | 13.3 | 13.6 |
| 40-60 | 4.2 | 27.0 | 19.1 | 17.1 | 12.8 | 13.4 | 17.1 | 14.5 | 11.6 | 12.3 | 21.3 | 19.5 | 12.9 | 12.7 |
| 60-80 | 2.7 | 22.9 | 17.4 | 12.8 | 11.2 | 11.4 | 15.8 | 13.8 | 10.5 | 11.0 | 17.1 | 16.5 | 11.8 | 11.5 |
| 80-100 | 1.9 | 16.5 | 15.3 | 10.4 | 9.5 | 8.8 | 12.3 | 11.5 | 9.4 | 8.6 | 14.3 | 13.7 | 9.4 | 9.0 |

2016

| Nmin | CK | MF | S25 | S50 | S75 | S100 | CM25 | CM50 | CM75 | CM100 | PM25 | PM50 | PM75 | PM100 |
| --- | --- | --- | --- | --- | --- | --- | --- | --- | --- | --- | --- | --- | --- | --- |
| 0-20 | 3.6 | 18.2 | 18.8 | 17.2 | 16.6 | 15.4 | 17.6 | 17.3 | 13.7 | 14.3 | 20.0 | 19.2 | 12.7 | 13.1 |
| 20-40 | 4.7 | 23.3 | 22.3 | 21.3 | 18.5 | 18.2 | 22.1 | 18.9 | 15.8 | 14.4 | 22.9 | 20.8 | 16.0 | 14.3 |
| 40-60 | 4.0 | 25.6 | 22.9 | 20.6 | 16.7 | 17.4 | 22.3 | 18.2 | 11.0 | 11.7 | 23.4 | 22.5 | 13.3 | 12.0 |
| 60-80 | 2.6 | 24.0 | 13.9 | 12.2 | 10.7 | 10.9 | 15.0 | 13.1 | 8.9 | 8.8 | 17.7 | 15.6 | 11.7 | 10.9 |
| 80-100 | 1.8 | 17.0 | 13.0 | 9.9 | 9.1 | 8.3 | 11.7 | 10.9 | 8.0 | 8.6 | 14.0 | 13.0 | 8.9 | 8.5 |
